# Strength after the arthroscopic Latarjet procedure: Are shoulder internal rotation, elbow flexion & supination strength decreased?

**DOI:** 10.1177/17585732231165227

**Published:** 2023-03-23

**Authors:** Naser Alnusif, Ali Lari, Saad AlQahtani, George S Athwal

**Affiliations:** 1 St Joseph's Health Care, Hand and Upper Limb Centre, University of Western Ontario, London, Ontario, Canada; 2 Department of Orthopedic Surgery, AlRazi Orthopedic Hospital, Kuwait City, Kuwait; 3 Department of Orthopedic Surgery, King Fahad Hospital of the University, 48023 Imam AbdulRahman Bin Faisal University, Al-Khobar, Saudi Arabia

**Keywords:** Latarjet, arthroscopic Latarjet, isokinetic, shoulder instability, strength, shoulder, elbow

## Abstract

**Background:**

The Latarjet procedure is an effective shoulder stabilizing surgery, however, the procedure results in an alteration of anatomy that may result in shoulder and elbow weakness. Thus, the purpose of this study was to assess post-operative shoulder and elbow strength after the Latarjet procedure. We hypothesized that shoulder and elbow strength are not affected after the procedure.

**Methods:**

The study group consisted of patients that had undergone the arthroscopic Latarjet procedure. An isokinetic dynamometer was used to evaluate the strength of bilateral shoulder internal rotation, elbow flexion, forearm supination using peak torque (N/m), as well as grip strength (kilograms). Shoulder range of motion and the potential effects of hand dominance were further analysed.

**Results:**

Nineteen patients with a mean age of 29 years and an average follow up of 47 months were included. Shoulder internal rotation strength, elbow flexion and forearm supination strength and grip strength were not significantly different when compared to the non-operative side (*p* > 0.13). The range of shoulder external rotation was significantly reduced (*p* < 0.001) on the Latarjet side.

**Conclusion:**

The results from this study demonstrate no statistically significant differences in the strength of shoulder internal rotation, elbow flexion, forearm supination or grip strength despite the surgical alterations to the subscapularis and conjoint tendon.

## Introduction

Anterior shoulder instability is common, with an estimated incidence of 1–2% of athletic injuries.^1,2^ Instability is often complicated by glenoid and humeral bone loss, capsular laxity and capsulo-labral insufficiency.^3^ The management options for anterior shoulder instability are usually influenced by patient factors, the magnitude of bone loss and the location and degree of soft tissue disruption.

In the presence of critical glenoid bone loss, which is in itself an evolving and controversial definition, the Latarjet procedure has been identified as an effective surgical treatment option, particularly in young and active patients. The main anatomical alterations that result from this procedure include splitting the subscapularis muscle to pass the coracoid bone block, which in turn leads to tensioning of the lower subscapularis creating a sling effect. The transfer of the coracoid bone block leads to alteration of the conjoint tendon insertion, which may alter the anatomic course of the musculocutaneous nerve.

As such, the resting length and line-of-pull of the short head of the biceps and the coracobrachialis are potentially changed. These anatomical changes could potentially affect shoulder or elbow strength and/or range of motion of the muscles involved. In the senior authors experience, a common question among patients indicated for the Latarjet procedure after it is explained to them, is whether there is any associated biceps weakness post-operatively. As such, the purpose of this study was to investigate whether the resultant anatomical alteration of the conjoint tendon insertion results in any decreases in forearm supination and/or elbow flexion strength. This was investigated with an isokinetic dynamometer machine. In addition to strength, patients at final follow-up were also assessed for range of motion, American Shoulder and Elbow Surgeons (ASES) score, and Western Ontario Shoulder Instability (WOSI) score.

## Materials and methods

### Study design and population

This was a single-centre retrospective analysis that included patients that had undergone an arthroscopic Latarjet procedure for anterior shoulder instability between March 2014 and January 2018 (3.8-year period) by a single surgeon (G.S.A). Informed consent was obtained from eligible patients prior to their inclusion in the study. Ethical approval was obtained from Lawson Health Research Institute (Approval number: R-18-109).

Eligible participants were over 18 years of age that had undergone an arthroscopic Latarjet procedure for anterior shoulder instability. Participants were invited to participate in the study at least one year postoperatively. Exclusion criteria included: any contralateral shoulder surgery, complex ipsilateral injuries that would affect shoulder function and previous Latarjet procedures.

During the aforementioned period, 78 arthroscopic Latarjet procedures were performed. All patients were contacted on three separate occasions, 30 patients were unreachable, 18 patients refused to participate on account of living distance, 11 patients excluded due to incomplete follow-up, leaving a total of 19 included participants.

### Surgical procedure

The arthroscopic Latarjet procedure^4^ was performed with patients under general anaesthesia in the beach chair position. The procedure begins by an initial diagnostic joint evaluation and exposure via the standard posterior and anterior central portals. The antero-inferior glenoid labrum is debrided as required, followed by electro-cautery marking of the graft placement roughly between the 2 o’clock and the 5 o’clock positions. Next, through the antero-lateral portal, the base of the coracoid and the conjoint tendon are visualized, and superior and lateral tissues are cleared. The axillary nerve is visualized along with the musculocutaneous nerve after pectoralis minor and coracoacromial ligament release.

Under direct vision, the subscapularis is split longitudinally between about 40–50% height from inferior to superior. After coracoid preparation, two guide wires are placed through the coracoid from an accessory portal, followed by drilling and tapping. The coracoid is then osteotomized, passed through the split, and pinned to the anterior glenoid vault between the 2 o’clock and 5 o’clock positions. A cannulated screws system is then used to arthroscopically secure the coracoid graft to the glenoid with two 4.5 mm cancellous threaded screws.

Postoperative rehabilitation protocol consisted of resting the arm in a sling for the first 0–2 weeks. At 2 weeks pendulum exercises were initiated. Next, active assisted range of motion exercises began at 2–4 weeks, followed by active range of motion exercises from 4–6 weeks onwards. Strengthening exercises were initiated at 3 months. It is the senior author's practice to allow unrestricted activity and strengthening at 3 months postoperative, which allowed for a minimum of 9 months of strengthening before assessment.

## Testing methods

### Clinical evaluation and outcome variables

Outcome variables included bilateral shoulder range of motion and strength of shoulder internal rotation, elbow flexion, forearm supination and grip strength. Additionally, patient age, sex, hand dominance, glenoid bone loss and follow-up period were recorded. Glenoid bone loss was measured by computed tomography on the sagittal view using the best-fit circle and measuring the percentage of bone loss.

Participants were evaluated clinically to assess bilateral shoulder range of motion. Shoulder forward elevation, external rotation and abduction were measured by use of a standard long arm goniometer. Participants were further assessed using the ASES and the WOSI scores. Results were subsequently documented and compared in the final analysis.

### Isokinetic assessment

Isokinetic evaluation was performed using the Biodex System 3 PRO isokinetic dynamometer (Biodex Medical Systems, NewYork, USA). Assessment included bilateral elbow and shoulder strength. To test shoulder internal rotation strength, patients were seated, with their shoulder in 45° abduction, elbow at 90° flexion and forearm in neutral pronation/supination. The trunk was stabilized by a belt to limit external forces. Next, to test elbow flexion and forearm supination strength, the shoulder was positioned in adduction. Elbow flexion was performed with the forearm in full supination. Grip strength was assessed using a handheld dynamometer with the forearm in neutral pronation/supination, with the elbow flexed at 90°.

The concentric/eccentric mode was used to assess the peak torque force. The assessment protocol for strength consisted of five cycles of each test, with a 1-min recovery period between each cycle followed by repeating the same steps on the contralateral side. For strength, the best result from the five cycles was used. The strength of shoulder internal rotation, elbow flexion and forearm supination were measured in peak torque (N-m; Newton/meter), while grip strength was measured in kilograms (kg) of force produced.

### Statistical analysis

Statistical analysis was performed using R v 3.6.3 (R Foundation for Statistical Computing, Vienna, Austria). Counts and percentages were used to summarize the distribution of categorical variables. The mean ± standard deviation (SD) and the median/interquartile range [IQR] were used to summarize the distribution of continuous normal and non-normal variables, respectively. Linear regression was used to assess the association between the observed difference and injury side in relation to hand dominance (same vs. different side). Paired *t*-test was used to assess whether there was a difference in various parameters between the affected and unaffected shoulders. The difference and the corresponding 95% confidence intervals were calculated. Hypothesis testing was performed at a 5% level of significance.

## Results

The study sample included 19 patients (21% females and 79% males) (Table 1). The average age of the patient group was 29 ± 10 years. The left side was injured in 58% of the patients. The injury occurred in the dominant hand in 42% of the cases. Average glenoid bone loss was 16% ± 6%.

**Table 1.** Descriptive statistics for the study group.

	** [ALL]**
	*** n = 19***
**Gender:**	
Female	4
Male	15
**Age (**mean ± SD**)**	29 ± 10
**Injured side:**	
Left	11
Right	8
**Hand dominance:**	
Left	2
Right	17
**Dominant sided injury:**	
No	11
Yes	8
**Glenoid bone loss** % (mean ± SD)	16 ± 6
**Follow-up period –** months (mean ± SD)	47 ± 17
** Patient reported outcomes**	
ASES (mean, range)	89 ± 13 (53–100)
WOSI (mean, range)	26 ± 24 (1–81)

ASES: American Shoulder and Elbow Surgeons Shoulder Score; WOSI: Western Ontario Shoulder Instability Score.

**Figure 1.**
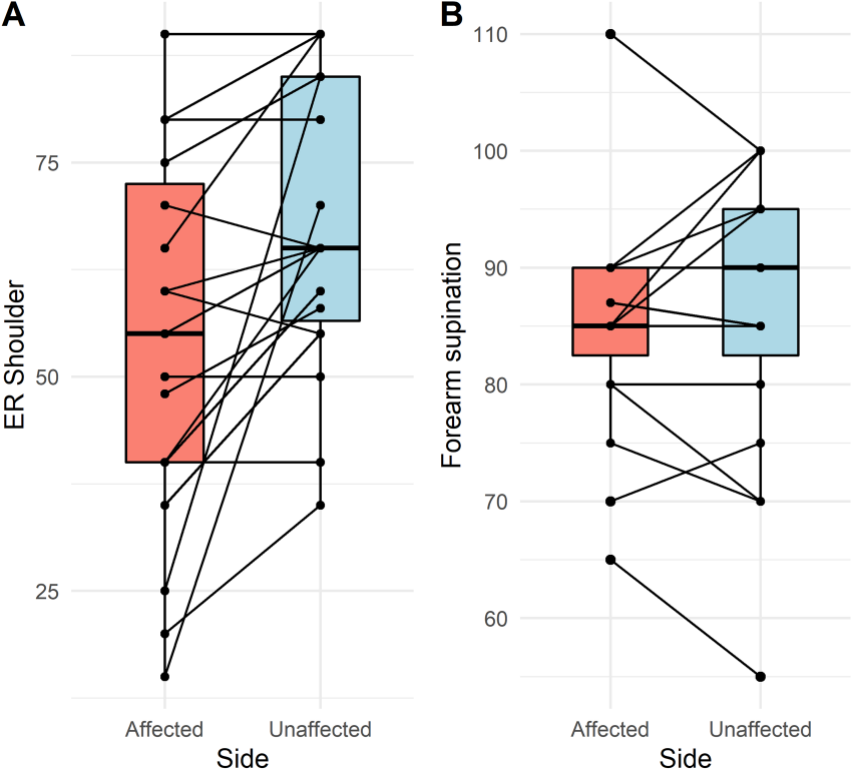
Difference in (A) external rotation range of motion (degrees) and (B) forearm supination strength (peak torque) between Latarjet and contralateral sides.

**Figure 2.**
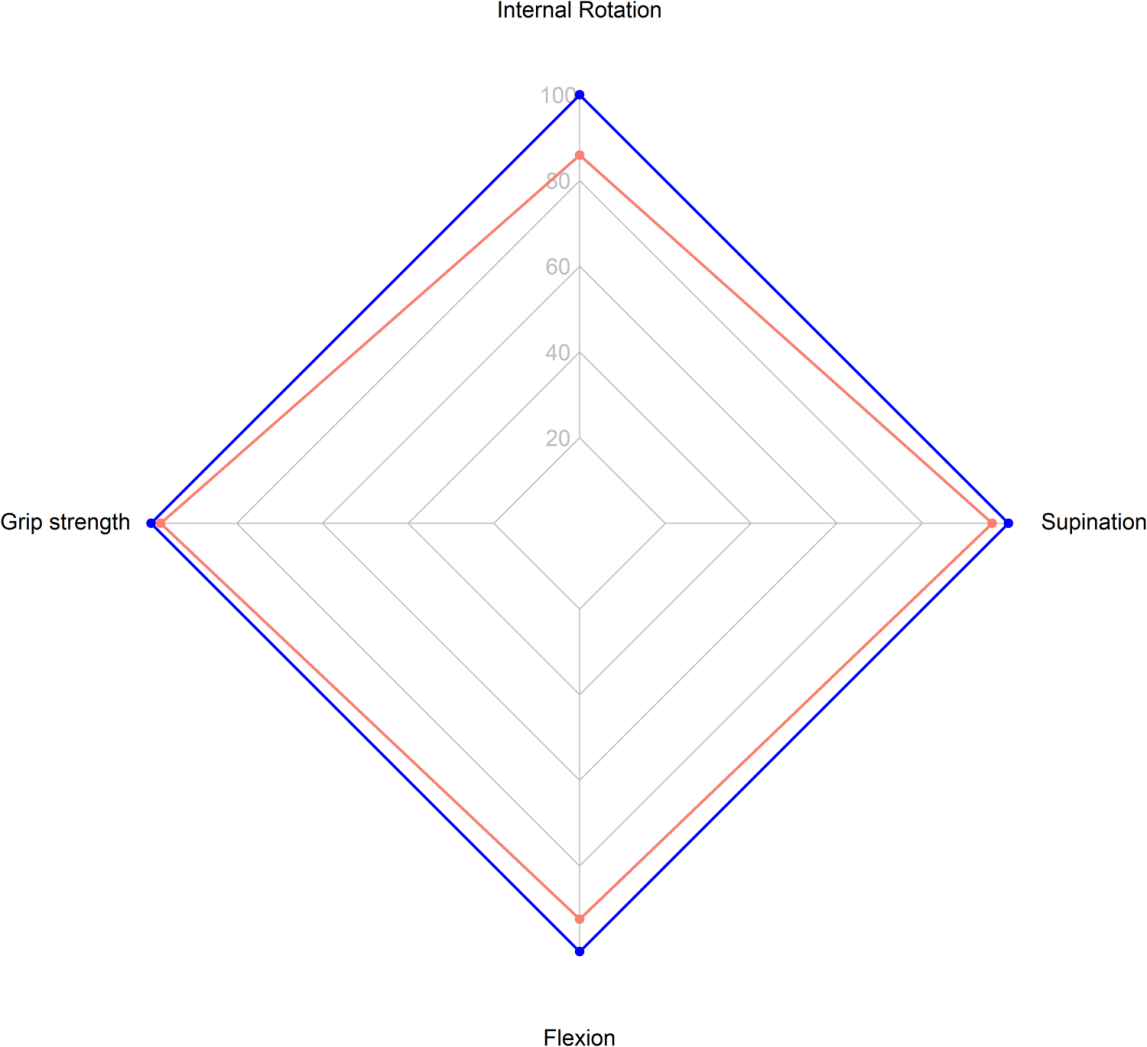
Radar chart for the ratio of shoulder internal rotation strength, grip strength, elbow flexion and supination strength between Latarjet (red) and contralateral sides (blue).

When comparing the Latarjet side to the unaffected side (Table 2), there were no statistically significant differences between any of the variables measured, other than for external rotation range of motion, which was less after the Latarjet (Δ = −14, *p* < 0.001) (Figure 1). The differences in strength are further summarized in (Figure 2).

**Table 2.** Comparison between the affected (Latarjet) and unaffected sides.

	**Latarjet**	**Contralateral**	**Δ**	**95% CI**	***p-*value**
**Shoulder ROM (degrees)**					
Shoulder flexion	161 (14)	162 (15)	−0.6	−3.213;1.95	0.61
Shoulder abduction	151 (26)	156 (23)	5	−11.017;1.227	0.11
Shoulder ER	54 (22)	68 (17)	−14	−22.584;−5.311	**< 0.001**
**Hand grip strength and shoulder internal rotation strength**					
Average hand grip strength (kg)	49 (15)	48 (14)	0.74	−1.307;2.781	0.46
Internal rotation (peak torque)	29 (30)	30 (22)	−1.32	−8.006;5.374	0.68
**Elbow flexion and forearm supination strength**					
Elbow flexion (peak torque)	35 (24)	37 (25)	−1.74	−4.251;0.777	0.16
Forearm supination (peak torque)	6.7 (4)	6.0 (3)	0.68	−0.211;1.579	0.13

Continuous variables were summarized using mean ± SD. Statistical analysis was performed using paired *t*-test.

With regards to hand dominance, results showed that the difference in average grip strength between the affected (Latarjet) and unaffected sides can be explained by the relation of hand dominance and the injured side. When the Latarjet side was dominant, the strength difference was lower (*p* = 0.016). However, none of the remaining comparisons were statistically significant, suggesting that there was no significant effect of dominance on the shoulder and elbow strength comparisons (*p* = 0.857).

## Discussion

The results from this study demonstrate comparable shoulder internal rotation and forearm supination strength bilaterally after the arthroscopic Latarjet procedure. This suggests that the anatomical alterations caused by the conjoint tendon transfer and the subscapularis split do not substantially affect the strength of the involved muscles. This is important, as patients indicated for the Latarjet procedure tend to be young, active and athletic. Additionally, it is important that professional athletes in contact or collision sports undergoing the Latarjet procedure understand that strength alterations after surgery are minimal. Interestingly, although side-to-side strength was similar, at follow-up, our patients had significantly less external rotation motion on the operative side (54 ± 22 vs 68 ± 17).

Overall, there are few isokinetic studies on the assessment of muscle strength after the Latarjet procedure. Caubere et al. described a series of 20 patients after open Latarjet procedures and reported a significant reduction of strength in internal and external rotation of the shoulder at 1-year follow-up.^5^ Edouard et al. performed an isokinetic assessment of 20 patients after open Latarjet and reported expected deficits in rotator cuff strength post-operatively, which normalized at 6 months.^6^ However, Ernstbrunner et al. reported reduced strength in internal and external rotation of the shoulder after open Latarjet surgery.^7^

In a sample of 20 patients, Malavolta et al. similarly evaluated shoulder and elbow strength after open Latarjet surgery, however, they reported reduced strength of external rotation and forearm supination, findings that differ from ours.^8^ In a sample of 20 patients undergoing open Latarjet surgery, Lacheta et al. reported reduced forearm supination strength but did not affect elbow flexion at a mean follow up of 9.8 months.^9^ However, studies assessing shoulder and elbow strength after arthroscopic Latarjet procedures were not found in the literature. Further, while all studies share similar methodology, small sample sizes and heterogeneity among groups may be potential causes of the differing results.

The influence of hand dominance on upper extremity strength is controversial. Nevertheless, hand dominance may influence strength.^10^ An analysis of our results indicated that hand dominance was a significant factor in governing strength in the dominant limb, regardless of the side of the injury. The influence of hand dominance was especially true in overall grip strength, as the results substantially favoured the dominant side.

The actual loss of strength or the thought that strength may be reduced after surgery has an influence on patient expectations. Patients, for obvious reasons, want a complete return of range of motion and strength. The restoration of function although important for all patients, may be especially important for high functioning athletes. The Latarjet procedure has been described as a non-anatomic procedure that alters the location of the conjoined tendon and disrupts to varying degrees the subscapularis muscle and tendon. Fortunately, our results indicate that the anatomic alteration of the conjoint tendon does not result in substantial changes in elbow flexion or supination strength. Further, the patient reported outcomes measured by the ASES and WOSI scores in our study were favourable and similar to those reported in previous literature.^11,12^

This study does have limitations. First, the time point for the assessment of postoperative strength varied between patients. However, all patients were a minimum of 1 year postoperative. Additionally, our sample size was relatively small, mainly due to the inability to predictably get this younger patient population to return to clinic for examination and assessment of strength. In many cases, we were unable to connect with patients, as the research ethics board only allowed a restricted number of contact phone calls and messages per patient. Finally, the results of this study are specific to the arthroscopic Latarjet procedure, however, the open and arthroscopic Latarjet have very similar anatomic alterations and we believe our results can be generalized to the open Latarjet procedure.^13^

## Conclusion

The Latarjet procedure is an effective shoulder stabilizing surgery, however, it results in alteration of anatomy that is questioned to result in shoulder and elbow weakness. The results of this study indicate that after the arthroscopic Latarjet procedure, shoulder internal rotation strength, elbow flexion strength, forearm supination strength and grip strength are not significantly different than the contra-lateral normal side. The findings of this study suggest that the Latarjet procedure more commonly affects range of motion rather than shoulder and elbow strength.

## Supplemental Material

sj-docx-1-sel-10.1177_17585732231165227 - Supplemental material for Strength after the arthroscopic Latarjet procedure: Are shoulder internal rotation, elbow flexion & supination strength decreased? Supplemental material, sj-docx-1-sel-10.1177_17585732231165227 for Strength after the arthroscopic Latarjet procedure: Are shoulder internal rotation, elbow flexion & supination strength decreased? by Naser Alnusif, Ali Lari, Saad AlQahtani and George S Athwal in Shoulder & Elbow

sj-docx-2-sel-10.1177_17585732231165227 - Supplemental material for Strength after the arthroscopic Latarjet procedure: Are shoulder internal rotation, elbow flexion & supination strength decreased? Supplemental material, sj-docx-2-sel-10.1177_17585732231165227 for Strength after the arthroscopic Latarjet procedure: Are shoulder internal rotation, elbow flexion & supination strength decreased? by Naser Alnusif, Ali Lari, Saad AlQahtani and George S Athwal in Shoulder & Elbow
