# Supplementary material for: Strength after the arthroscopic Latarjet procedure: Are shoulder internal rotation, elbow flexion & supination strength decreased?
Source: Shoulder Elbow. 2023 Mar 23;16(1):53–8. doi: 10.1177/17585732231165227 (PMC10902414; doi:10.1177/17585732231165227)
Supplement: sj-docx-1-sel-10.1177_17585732231165227 - Supplemental material for Strength after the arthroscopic Latarjet procedure: Are shoulder internal rotation, elbow flexion & supination strength decreased? [file sj-docx-1-sel-10.1177_17585732231165227.docx]

| **Patient no.** | **ASES Score** | **WOSI Score** |
| --- | --- | --- |
| 1 | 100 | 1 |
| 2 | 95 | 15 |
| 3 | 93 | 3 |
| 4 | 83 | 60 |
| 5 | 100 | 4 |
| 6 | 95 | 16 |
| 7 | 95 | 16 |
| 8 | 100 | 10 |
| 9 | 58 | 72 |
| 10 | 95 | 36 |
| 11 | 97 | 28 |
| 12 | 53 | 81 |
| 13 | 90 | 16 |
| 14 | 75 | 22.00 |
| 15 | 80 | 50 |
| 16 | 100 | 4 |
| 17 | 100 | 5 |
| 18 | 97 | 21 |
| 19 | 90 | 33 |

**Supplementary Table 1**. Individual case data for the American Shoulder and Elbow Score (ASES) and Western Ontario Shoulder Instability Index (WOSI).
