# Supplementary material for: Strength after the arthroscopic Latarjet procedure: Are shoulder internal rotation, elbow flexion & supination strength decreased?
Source: Shoulder Elbow. 2023 Mar 23;16(1):53–8. doi: 10.1177/17585732231165227 (PMC10902414; doi:10.1177/17585732231165227)
Supplement: sj-docx-2-sel-10.1177_17585732231165227 - Supplemental material for Strength after the arthroscopic Latarjet procedure: Are shoulder internal rotation, elbow flexion & supination strength decreased? [file sj-docx-2-sel-10.1177_17585732231165227.docx]

Supplementary File 1. Association between observed difference and injured side (unaffected – affected) in relation to hand dominance

**

Each row represents the association between the injury side in relation to hand dominance and the observed difference in effect. Confidence intervals which included zero are not statistically significant at the 0.05 level; IV: Independent variable

Results showed that the observed difference in average grip strength can be explained by the relation between affected and dominant sides. The difference in the average grip strength was lower by 4.56 (B = -4.56, p = 0.016) when the affected side was the dominant one indicating that the difference is lower when the affected side is the dominant one.
